# Rainfall, temperature, and Classic Maya conflict: A comparison of hypotheses using Bayesian time-series analysis

**DOI:** 10.1371/journal.pone.0253043

**Published:** 2021-07-30

**Authors:** Mark Collard, W. Christopher Carleton, David A. Campbell

**Affiliations:** 1 Department of Archaeology, Simon Fraser University, Burnaby, Canada; 2 Extreme Events Research Group, The Science of Human History, and Biogeochemistry, Max Planck Institutes for Chemical Ecology, Jena, Germany; 3 School of Mathematics and Statistics, Carleton University, Ottawa, Canada; University at Buffalo - The State University of New York, UNITED STATES

## Abstract

Studies published over the last decade have reached contrasting conclusions regarding the impact of climate change on conflict among the Classic Maya (ca. 250-900 CE). Some researchers have argued that rainfall declines exacerbated conflict in this civilisation. However, other researchers have found that the relevant climate variable was increasing summer temperatures and not decreasing rainfall. The goal of the study reported here was to test between these two hypotheses. To do so, we collated annually-resolved conflict and climate data, and then subjected them to a recently developed Bayesian method for analysing count-based times-series. The results indicated that increasing summer temperature exacerbated conflict while annual rainfall variation had no effect. This finding not only has important implications for our understanding of conflict in the Maya region during the Classic Period. It also contributes to the ongoing discussion about the likely impact of contemporary climate change on conflict levels. Specifically, when our finding is placed alongside the results of other studies that have examined temperature and conflict over the long term, it is clear that the impact of climate change on conflict is context dependent.

## Introduction

Concern is growing among policy-makers that anthropogenic climate change will lead to an increase in conflict [1–3]. However, the scientific literature is far from clear about the likelihood of this outcome and the specific mechanisms involved. Some researchers have found that rising temperatures are associated with increased conflict [e.g., 4], while others have failed to find any effect of increasing temperatures on conflict levels [e.g., 5]. Still others have concluded that cooler rather than warmer temperatures lead to increased conflict [e.g., 6, 7]. The effect of rainfall on conflict is equally unclear. Some studies have found that greater rainfall variability (i.e., more frequent or pronounced dryness or wetness) increases conflict onset frequency [e.g, [8] or contributes to the persistence of existing conflict [9], while others have concluded that such variability, especially if it leads to moisture shortages sufficient to impact agriculture, reduces conflict [e.g., 10, 11]. Even reviews of the literature covering dozens of individual studies have failed to clarify the situation, reporting instead highly heterogeneous results [e.g., 8, 12–15]. Given this, it is perhaps unsurprising that a recent survey found that most scholars working on the topic think that much remains uncertain about the impact of climate change on conflict [16]. The continuing uncertainty has led to calls for researchers to increase the diversity of case studies [17], and to investigate the specific environmental, sociocultural, and economic factors that mediate the relationship between climate change and conflict [18].

**Fig 1:**
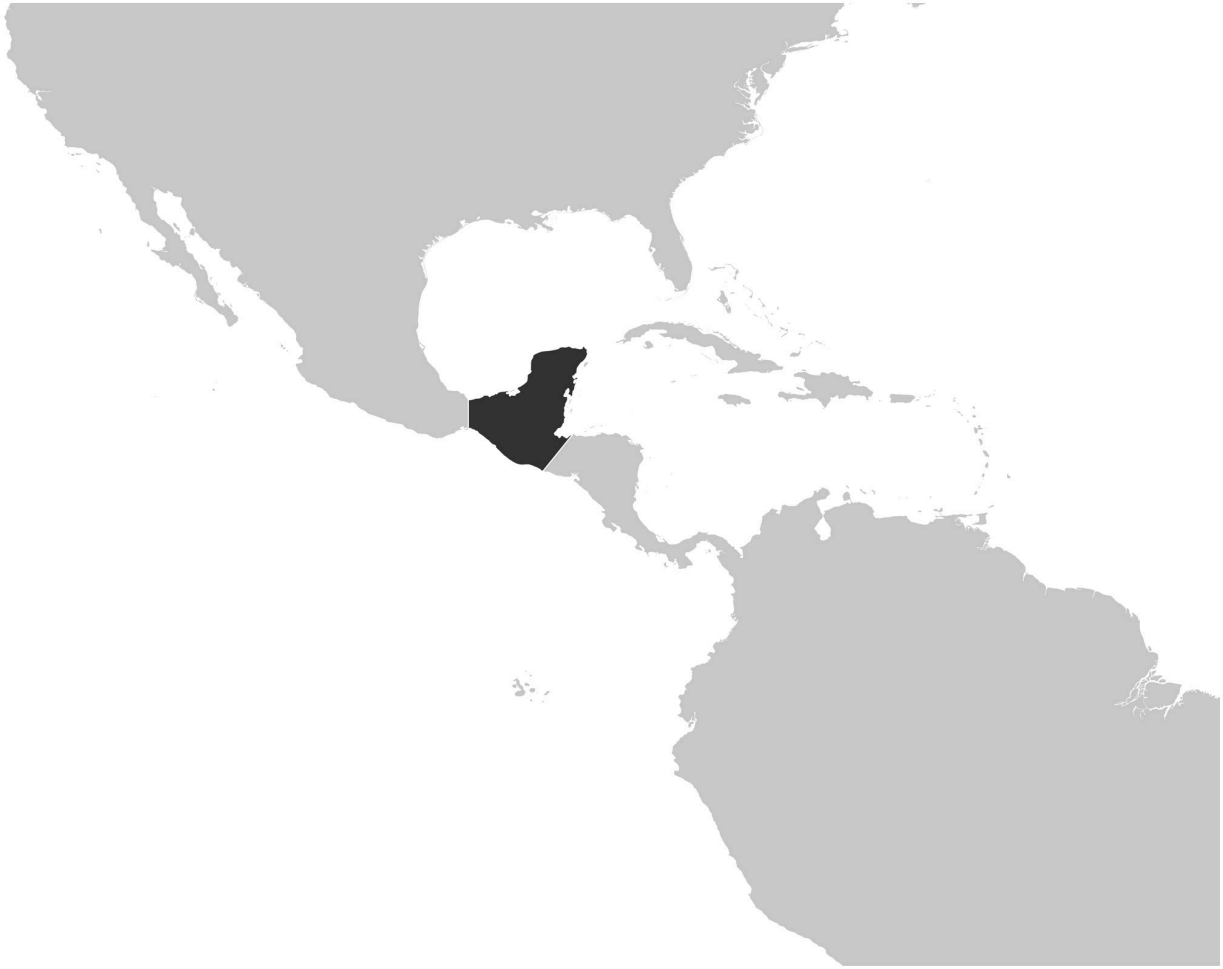
Map showing the geographic location of the Classic Maya. The dark grey area roughly corresponds to the Classic Maya region.

Partly with these calls in mind, we conducted a study that sought to shed light on the relationship between climate change and violent inter-group conflict in a well-known historical civilisation—the Classic Maya. The Maya are indigenous people who occupy a region close to the middle of the isthmian portion of North America (Fig 1). Scholars typically consider the Classic Period of Maya history to have begun in 250 CE and ended by around 900 CE in the southern lowlands with some continuity in other regions until around 1050 CE [19]. The period is often further subdivided into the Early Classic (250–600 CE) and Late Classic (600–900 CE) on the basis of changes in art and architectural styles that appear to be associated with a major cultural and political shift [20] with some scholars including a final “Terminal Classic” stage from 750–1050 CE [21]. The Classic Maya are noteworthy for having constructed large cities featuring pyramids and other monumental buildings. They also developed one of the few writing systems in the Americas and a precise calendar [22, 23]. It is clear from translated inscriptions and surviving artwork that violent conflict was an important feature of Classic Maya life [24–27]. Typically, the conflicts were a manifestation of political competition between divine kings who ruled over city-states. Among the conflict events mentioned in the epigraphic record are captive-taking raids, attacks to demand tribute, and large coordinated offensives that appear timed to coincide with astronomical events and have therefore been dubbed “star wars” [27].

Recent studies have reached contrasting conclusions regarding the impact of climate change on Classic Maya conflict. Kennett et al. [28] compared the epigraphic record of Classic Maya conflict and an oxygen isotope (*δ*^18^O) rainfall proxy from Yok Balum Cave, Belize, and found an association between declining rainfall and increased conflict. They argued that this association was caused by the negative impact of rainfall shortages on the agricultural basis of Classic Maya society. Conflict levels increased, they suggested, because Maya polities fought over diminishing resources as rainfall levels declined throughout the Classic period. A few years later, Carleton et al. [29] arrived at a different conclusion. These authors used time-series analysis to compare the epigraphic record of Classic Maya conflict, the Yok Balum Cave *δ*^18^O rainfall proxy, and sea-surface temperature reconstructions from the Cariaco Basin. They found no association between rainfall variability and conflict. Instead, their analyses indicated that conflict was influenced by summer temperatures. Based on recent agricultural research [30], Carleton et al. [29] proposed that increasing average growing season temperatures during the Classic period led to declines in maize yields, and that these declines created crises of political legitimacy because of the symbolic association between Classic Maya rulership and maize. Crop failures were perceived as political failures that undermined the elite’s right to rule. In order to cope with this, Carleton et al. [29] suggested, members of the elite organized attacks on other polities, hoping that victory would restore that lost legitimacy. The main difference between the hypotheses, then, is the specific mechanism by which climatic change affected Classic Maya conflict levels. Testing between the hypotheses was the goal of the study reported here.

**Fig 2:**
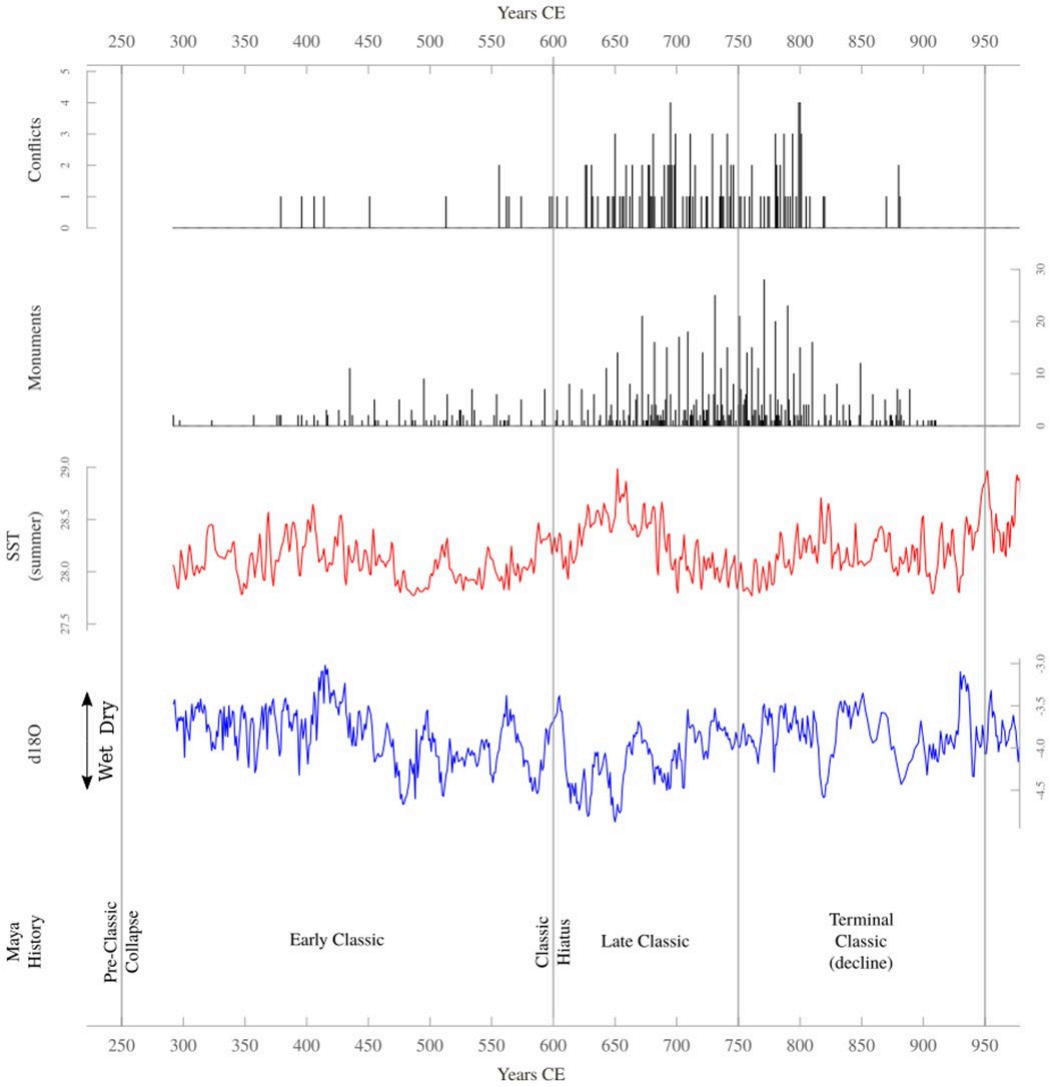
Time-series data and Classic Maya history.

Low temporal resolution is one potential explanation for the incongruent results of Kennett et al.’s [28] and Carleton et al.’s [29] analyses. Both teams relied on data with a resolution of 25 years, which potentially obscured considerable inter-annual variation. This, in turn, means it is possible that their studies did not capture real climate-conflict dynamics. With this in mind, we collated annually-resolved conflict and climate time-series for the period 292–997 CE (Fig 2), and then created a Bayesian time-series model for analysing count-based historic data. We then used the model to determine whether annually-resolved climatic variation could explain annually-resolved variation in conflict during the Classic period.

In the model, conflict at any given time is a function of conflict at all previous times and, optionally, one or more covariates. To account for the impact of previous levels of conflict, we included an autocorrelation term, which represents through-time persistence in the level of conflict. This term can be conceptualized in a number of ways, but when we decided to include it we had in mind the well-established phenomenon of retributive violence—i.e., the tendency for actors or groups to respond to violence with further violence or, conversely, to restrain themselves from violence when others have been peaceful. In line with several other scholars [e.g., 20, 27, 31], we suspect that much of the variation in Classic Maya conflict levels can be attributed to cycles of retributive violence. Temporal autocorrelation in conflict levels is also a well-known phenomenon in conflict research more generally [e.g., 32, 33]. So, we treated the autocorrelation process as the default or null process, and investigated whether the climate variables had an impact on conflict beyond that attributable to autocorrelation. Since the conflict data were derived from inscriptions on monuments, variation in the number of monuments erected and/or preserved could be a confounding factor. We accounted for this by modelling conflicts per monument, rather than simply the raw conflict counts. In our analysis, we compared four nested models using Bayes Factors to assess their evidential support:

- Retributive Violence—In this model, conflict levels were affected solely by autocorrelation in violence;
- Temperature—In this model, conflict levels were affected by both retributive violence and summer temperature variation;
- Rainfall—In this model, conflict levels were affected by both retributive violence and rainfall variation; and,
- Combined—In this model, conflict levels were affected by retributive violence, rainfall variation, and summer temperature variation.

Because Bayes Factors are relative measures of evidential strength, we adopted Jeffrey’s [34] framework for interpreting our results. According to this framework, Bayes Factor differences a lot lower than 1 indicate support for the baseline model, Retributive Violence; differences close to 1 indicate that we cannot distinguish between the tested climate model and Retributive Violence; differences between 1 and 5 indicate weak evidence favouring the climate model over Retributive Violence; differences between 10 and 15 indicate substantial evidence favouring the climate model over Retributive Violence; and differences greater than 15 indicate strong evidence favouring the climate model over Retributive Violence.

## Materials and methods

The dataset used in the study consists of annual values for the period 292–997 CE—i.e., covering most of the Classic and part of the terminal Classic period—for four variables (see S1 File). The first was the number of inter-polity conflicts per year. Values for this variable were obtained from several sources (see S1 File).

The second variable was the number of monuments erected each year. We included this variable because the conflict counts derive from inscriptions on monuments and we wanted to control for the potentially confounding effect of variation in the number of monuments erected. The values for this variable were taken from the same sources as the conflict data (see S1 File).

The third variable was an oxygen isotope (*δ*^18^O) record from Yok Balum Cave, Belize [28]. This was included as a proxy for variation in rainfall. While *δ*^18^O levels are known to be affected by both temperature and rainfall [35], Kennet et al. [28] found that variation in the Yok Balum Cave *δ*^18^O record was mostly caused by rainfall. They made this determination by conducting two analyses. One involved focused on a year’s worth of detailed rainfall and cave drip water observations at Yok Balum Cave; the other involved comparisons between the *δ*^18^O time-series and historic instrumental rainfall records. Both analyses revealed strong correlations between the *δ*^18^O record and regional rainfall amounts. Kennet et al. [28] also pointed out that other studies of *δ*^18^O records obtained in tropical regions have found that these records predominantly reflect rainfall variation [e.g., 35–38]. They argued that this is because temperatures do not vary greatly within or between years in the tropics and circulation in these regions is dominated by seasonal movement of the Inter-Tropical Convergence Zone (ITCZ). Together, Kennett et al. [28] averred, these climatic patterns tend to produce a clear rainfall signal in *δ*^18^O variation. Thus, there are reasons to believe that the Yok Balum Cave *δ*^18^O record adequately reflects variation in annual rainfall amounts—although recent research indicates that the record also reflects storminess to some extent [39]. The original *δ*^18^O record has a subannual resolution, so we created an annual record by calculating average values at one-year intervals using the core R function “spline” [40].

The last variable is a summer sea-surface temperature (summer SST) reconstruction based on Magnesium/Calcium (Mg/Ca) ratios derived from the shells of a planktonic foraminifera called *Globigerinoides ruber* (pink) contained in a sediment core from the Cariaco Basin [41]. The reconstruction reflects summer SST in the basin over the last 2000 years. Unlike some other foraminifera species in the basin, the abundance of *G. ruber* in the upper water column is not affected by Eckman upwelling—a process whereby circulation at the air–water interface brings deeper, colder waters to the surface. Upwelling in the Cariaco Basin is at its strongest during the winter season and is caused by seasonal southward migration of the ITCZ. During the summer season—when *G. ruber* peaks—the ITCZ is further north, upwelling is reduced, and the water column is more stratified [41]. Hence, Mg/Ca ratios in *G. ruber* shells reflect temperatures in the upper water column without the confounding effect of deeper, colder water mixing upward. Like other palaeoclimatic reconstruction techniques, palaeothermometry based on Mg/Ca ratios in foraminifera shells comes with various uncertainties and those need to be borne in mind (see [42–44]). Nevertheless, Wurtzel et al. [41] determined that their *G. ruber* temperature reconstruction correlates strongly with SST during the instrumental period in the Cariaco Basin. While the Cariaco Basin is quite far from the central Maya lowlands where most of the conflicts under analysis took place, Carleton et al. [29] found that the *G. ruber* Mg/Ca record correlates with other circum-Caribbean records, which suggests a region wide pattern of temperature change. They also found that the temperature reconstruction correlates with 20^th^ century air temperatures from the Petén Department of Guatemala where a major Classic Maya centre, Tikal, is located. As part of the present study, we evaluated the correlation between the Cariaco summer SST reconstruction and Climate Research Unit temperature records for the Maya region; our results were consistent with the ones reported in Carleton et al. [29] (see S1 File).

To determine which, if either, of the climate variables best explains the variation in conflict counts we created a new, Bayesian model for analysing count-based time-series. Carleton et al. [29] used the Poisson Exponentially-Weighted Moving Average (PEWMA) model [45] to assess the impact of climate change on Classic Maya conflict. However, we could not employ the PEWMA model in the present study because it cannot cope with long strings of zeros and there are several periods in our annually-resolved dataset, particularly in the Early Classic, when relatively few conflicts are mentioned.

**Fig 3:**
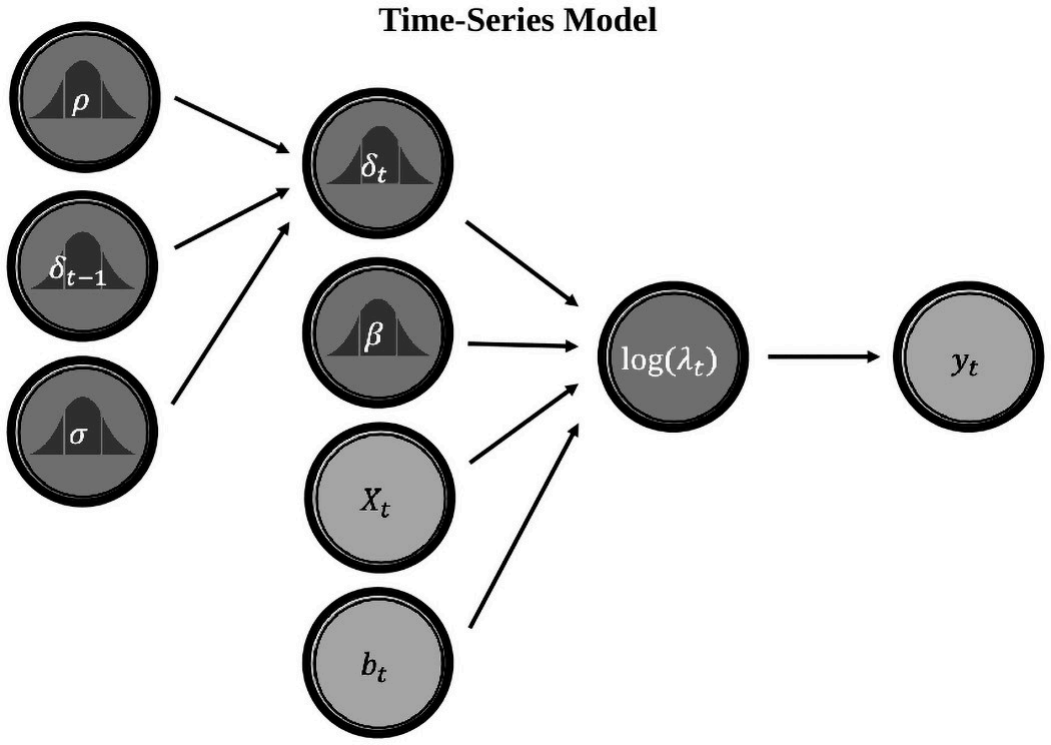
Directed acyclic graph for the time-series model. In this image, Light grey circles represent data—*y*_*t*_ refers to conflicts dated to a given time; *X*_*t*_ refers to a matrix of covariates; and *b*_*t*_ is the number of monuments dated to a given time. Dark grey circles represent terms estimated from the data (see 1–7). The dark grey density symbols indicate probabilistic terms, and terms without subscripts are non-time varying.

The model we developed is a flexible Bayesian state-space time-series model based on a model we recently used to study the climate–conflict relationship in second millennium Europe [46]. See Fig 3 for a visual schematic of the model. As the schematic indicates, state-space models are used to differentiate between observations and the process that produced them. The process is considered latent—i.e., it cannot be directly observed, and its parameters must instead be estimated based on a given set of observations. State-space models are defined by a set of equations. One equation represents the dependent variables that are actually observed, while another represents the unobserved latent process, which is affected by the independent covariates.

To analyse our Classic Maya conflict record, we assumed that the observed conflict time-series was produced by an unobservable conflict-generating process. Since the observed conflict time-series is count-based, and there is no such thing as fractional or negative conflict, an appropriate basis for the model is the Poisson distribution. Thus, the number of conflicts per period can be thought of as a series of Poisson-distributed random variables, denoted *Y* = [*y*_1_, *y*_2_.…, *y*_*t*_] where *t* is the number of periods. The conflict-count variables are therefore modelled as follows:

$$y_{t}∼P(\lambda_{t})$$ (1)

In this equation, *y*_*t*_ is the number of conflicts at time *t*, and λ_*t*_ is the mean of the Poisson-distributed process. The mean of the conflict-count process is, in turn, determined by the latent, unobservable, conflict process, which includes external inputs in the form of covariates. This latent process is represented by the following equation:

$$\lambda_{t}=b_{t}e^{X_{t}\beta +\delta_{t}}$$ (2)

In Eq 2, *b*_*t*_ is the number of monuments dated to time *t*. This term was included to control for the potentially confounding effects of variation in the number of monuments erected per year. It also implies the model can be interpreted as estimating the average number of conflicts per monument. The next two terms are a matrix of (mean-centered) covariates including an intercept (column of 1’s), *X*_*t*_, and a vector of regression coefficients, *β*, both related to the Poisson mean by a log-link function, which is why they are exponents of *e*. The last term, *δ*_*t*_, is also an exponent of *e* and it represents the underlying, endogenous autocorrelated conflict process. By including this term, we accounted for the observable autocorrelation in the conflict record (Fig 1 in S1 File), which seems likely to be due, at least in part, to cycles of retribution. To model these dynamics, we used an autocorrelated normally-distributed process,

$$\delta_{t}∼N(\rho \delta_{t-1},\sigma )$$ (3)

where the mean is represented by *ρδ*_*t*−1_ and the standard deviation is represented by *σ*. The mean, *ρδ*_*t*−1_ has two parts. The first, *ρ*, is an autocorrelation term; the second, *δ*_*t*−1_, is the level of the endogenous conflict process at the previous time (i.e., t-1). The autocorrelation term applies a weight to the influence of past conflict counts on present and future conflict. A positive value for *ρ* implies that endogenous conflict is increasing over time because the influence of past conflict is accumulating whereas a negative value implies it is declining because the influence is diminishing. The standard deviation of the distribution, *σ*, determines the volatility of the endogenous autocorrelated conflict process. Higher *σ* indicates higher magnitude fluctuations in the endogenous conflict process while lower *σ* indicates lower magnitude fluctuations. Importantly, the way autocorrelation is included makes *δ*_*t*_ a Markov process. This means that the amount of conflict at any given time (*t*) is conditional on conflict in the previous time (*t* − 1), which is in turn conditional on the level of conflict in the time before that (*t* − 2), and so on (*t* − *n*). However, a single parameter in the model, *ρ*, accounts for the whole chain of conditional dependence.

It should be noted that the model intentionally separates retributive violence from external climatic perturbations. The autocorrelation process just described avoids feedback that would be produced by including covariate effects in the model’s autocorrelation structure. Effectively, perturbations to the conflict process caused by any covariates included in the model (e.g., climate variables) cannot explode into additional future conflict. This limits the potential for exaggerating covariate effects, but it is clearly an assumption of the model that could be explored in future research.

Because the model is Bayesian, several parameters have prior distributions. The priors include normal distributions for the regression coefficients (*β*) of Eq 2, the autocorrelation coefficient (*ρ*) of Eq 3, and the first estimate of the endogenous conflict process (*δ*_0_) in Eq 3. The last of these is the estimated level of conflict in the period immediately prior to the beginning of the conflict record. The priors we used were informed by reasonable assumptions about their likely impact on conflict given the scale of the climate covariates and the observed levels of conflict in the record—i.e., we used *weakly-informative priors*, which is considered best practice in applied contexts [47]. For instance, we chose variances for the priors applied to regression coefficients, *β*, that would limit predicted numbers of conflicts per monument to less than 5 on average with a low probability for higher values (see S1 File). While using much more agnostic priors would not likely change the posterior estimates for the model’s parameters, implausibly vague priors are known to undermine model comparisons involving Bayes Factors [48]. These three priors were parameterized as follows:

β∼N(0,1) (4)

ρ∼N(0,10) (5)

$$\delta_{0}∼N(0,10)$$ (6)

We also used a prior for the standard deviation, *σ*, in Eq 3. Instead of a normal distribution, however, we used an exponential one so that the value would be positive, which is a requirement for standard deviations. This prior was parameterised as follows:

σ∼Exp(0.7) (7)

All of the prior distributions were ultimately transformed by the data and likelihood for the model via Bayes Theorem into posterior estimates of the relevant parameters [49].

In our analysis, we compared four properly nested models. The first was a retributive violence-only model—i.e., Eq 2 with both climate regression coefficients, *β*, set to zero. The interpretation of this model is that conflict counts are only determined by past conflict and not by climate change. In the next two models we allowed one climate regression coefficient to vary while the other was set to zero. In one of these the regression coefficient for the *δ*^18^O rainfall record was allowed to vary, while in the other the coefficient for the summer SST record was allowed to vary. These models imply that conflict counts are the product of past conflicts and one of the relevant climate variables. For the the fourth and final model we allowed the regression coefficients for both the temperature and rainfall covariates to vary freely. According to this model, conflict counts are a product of past conflicts and both temperature and rainfall.

To compare the models, we used Bayes Factors [49]. The Bayes Factors indicate the weight of evidence for a model given the data relative to the baseline Retributive Violence model—higher Bayes Factors indicate better fitting models. We reasoned that in order for a given climate covariate to be considered important for explaining variation in Classic Maya conflict, the model in which its regression coefficient was allowed to vary had to have a higher Bayes Factor than the other models and a higher Bayes Factor than the Retributive Violence model. To estimate the Bayes Factors we employed the widely-used Savage-Dickey Ratio [48]. Given that Bayes Factors are known to be sensitive to prior distributions, we also ran a repeat analysis following the same procedure as just outlined but with wider—more agnostic—priors (see S1 File).

To estimate the posterior distributions of the parameters for each model and the posterior probabilities for the models themselves, we used a Markov-Chain Monte Carlo (MCMC) approach. Each MCMC simulation was run for 1,000,000 iterations the first 1000 of which were discarded as burn-in. We used the Geweke [50] statistic (see S1 File) and visual inspection of the MCMC trace plots to evaluate convergence of the MCMC chains.

The calculations were performed in R [40]. We used a newly developed Bayesian model estimation package called ‘Nimble’ [51] for the MCMC simulations. The code used in the study can be found on GitHub (https://github.com/wccarleton/mayaconflictannual).

## Results

The conflict count time-series indicates there was a substantial increase in conflict between the Early and Late periods (Fig 2). That this pattern is real and not simply an artifact of the epigraphic record is supported by archaeological data, including defensive architecture, settlement placement, weaponry, and skeletal remains [26, 27, 52–55]. These other lines of evidence also suggest that there was a through-time increase in conflict. The agreement between the epigraphic record and the archaeological data regarding the existence of an upward trend in conflict suggests that none of the biases that could potentially impact the epigraphic record (e.g., political agendas, changes in recording practices, etc) is severe enough to undermine its use as a proxy for past conflict levels, especially given that our analysis is primarily concerned with long-term trends.

**Fig 4:**
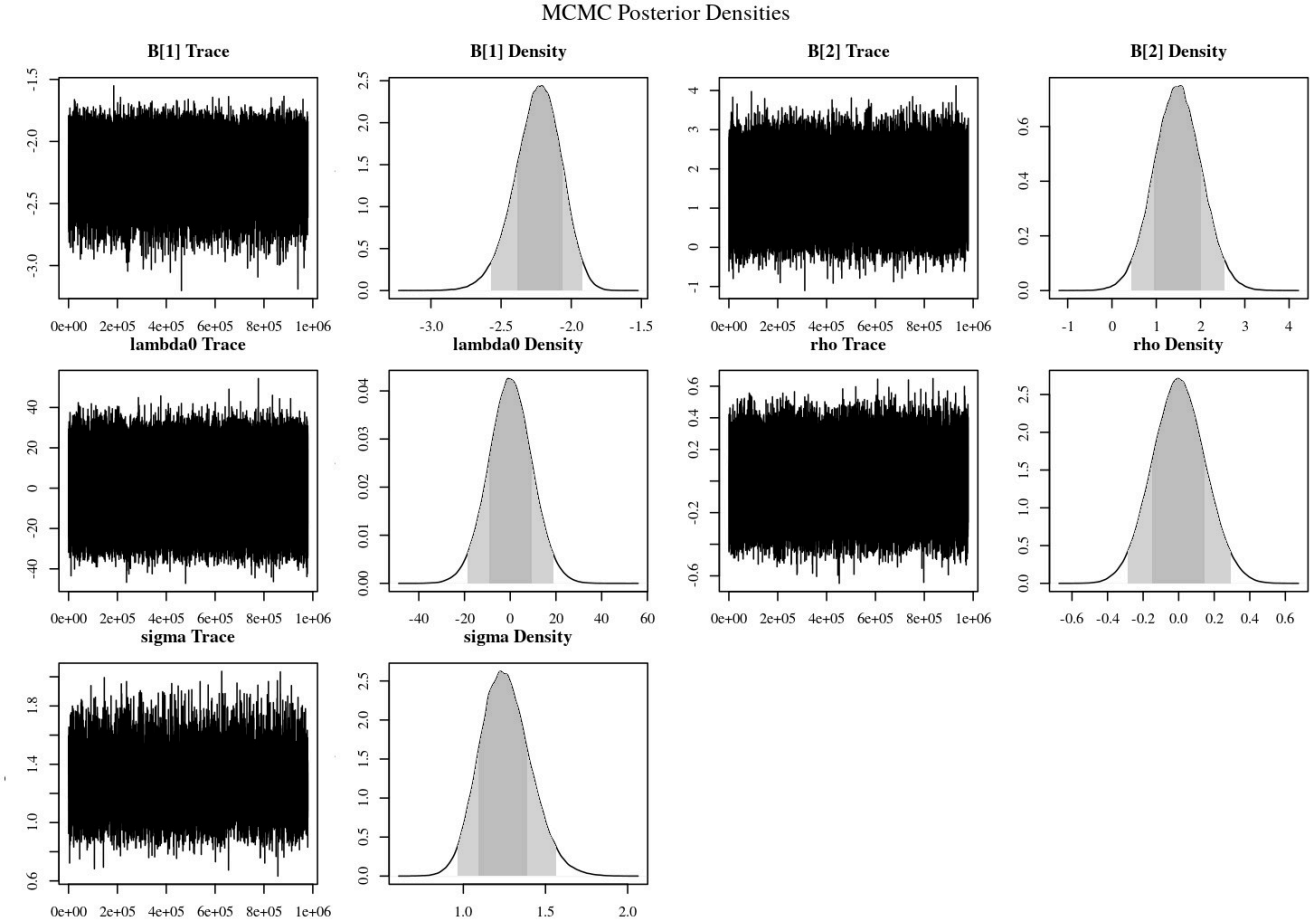
Posterior densities and MCMC trace plots for the Temperature model. The density functions contain two shaded regions: light grey corresponds to the 95% highest density region; and the dark grey corresponds to the 68% highest density region (see S1 File for similar plots corresponding to the other models).

Our results indicate that the Temperature model not only outperformed the other two climate models but also substantially outperformed the Retributive Violence model (Table 1). According to Jeffrey’s [34] framework, the evidence favouring the Temperature model is strong. The posterior density of the regression coefficient in Temperature indicates that there is a positive correlation between temperature and conflict—i.e., increased temperature is associated with increased conflict levels—and that the effect was substantial. On average there was a ∼16% increase in conflict per 0.1 C increase in temperature (see Fig 4).

**Table 1:** Model results. Rows represent the primary results of a single model.

Model	Bayes Factor	Temperature Coefficient	Rainfall Coefficient
Retributive Violence	1		
Temperature	21.68	1.49	
Rainfall	1.21		-0.61
Combined	10.62	1.39	-0.18

The first column indicates which if any climate covariates were included in a given model: ‘Retributive Violence’ indicates a retributive violence-only model; ‘Temperature’ a model containing the temperature covariate; and so on. The third column contains the Bayes Factors, which indicate the weight of evidence for each model in a given time period relative to the baseline, Retributive Violence model. The Bayes Factors can be straightforwardly interpreted as the number of times more probable a given model is than the baseline, Retributive Violence model. The last two columns contain the mean of the posterior density of the regression coefficient estimated for a given climate covariate.

## Discussion and conclusions

The analyses reported here suggest that the increase in conflict among Classic Maya polities between the Early and Late periods was influenced by growing-season temperature rather than by annual rainfall variation. This in turn implies that data resolution was indeed likely responsible for the disagreement between the results reported by Kennett et al. [28] and Carleton et al. [29]. As we mentioned earlier, both studies employed data-binning to produce a 25-year conflict time-series. While data-binning is unavoidable for count processes (because an observation period must be defined) in some contexts, the reduction in variation it entails can result in misleading biases. In this case, it appears that data-binning led Kennett et al.’s [28] analysis astray.

Our finding that higher summer temperatures exacerbated conflict is consistent with the hypothesis developed by Carleton et al. [29] to explain their results. To reiterate, their hypothesis argues that the impact of temperature on conflict among Classic Maya polities during the first millennium CE was mediated by the productivity of the Classic Maya’s staple crop, maize.

Carleton et al. [29] drew on agricultural research showing that temperature has a non-linear impact on maize yields [30]. According to this research, yields increase as temperature increases but only to 30°C. Above this temperature, yields decline by as much as 1% for each day spent above the threshold, even under optimal rainfall conditions. Other observational and experimental research involving multiple varieties of maize supports the idea that 30°C is the optimal temperature with respect to yield-affecting phases of maize development, and that higher temperatures have a deleterious effect on productivity, although the precise physiological mechanisms are still under investigation [56–61].

**Fig 5:**
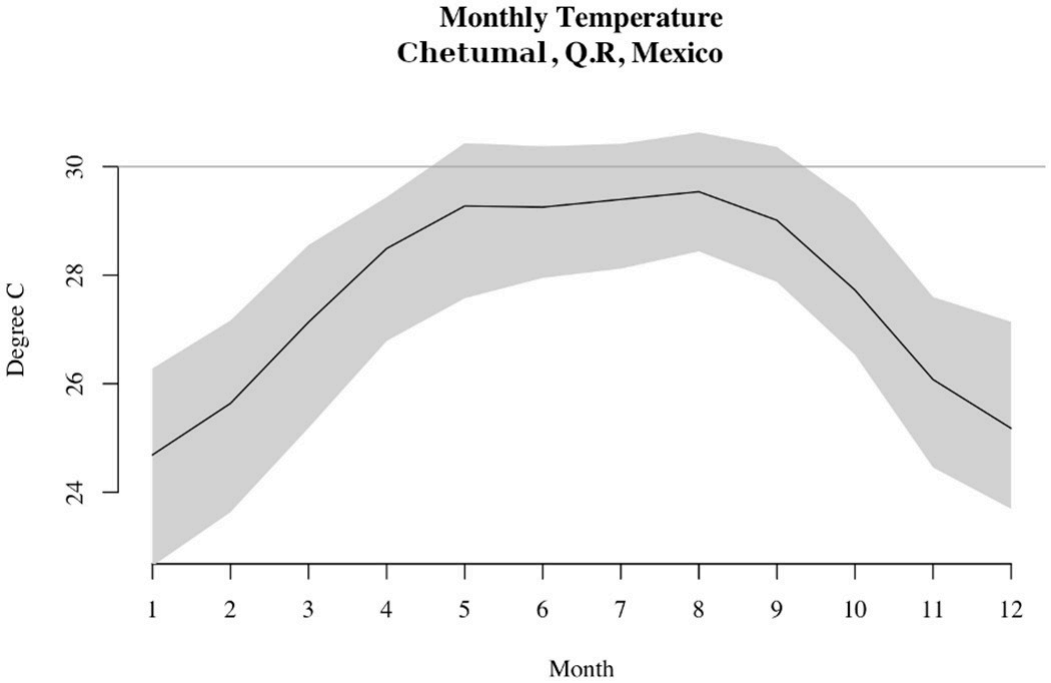
Climate Research Unit (CRU) temperature data. This plot shows monthly means, minimums, and maximums recorded by a weather station at Chetumal, Quintana Rue, Mexico—the nearest recording station to the Péten region of Guatemala where several major Classic Maya centres are located, e.g., Tikal. These temperature data were taken from http://climexp.knmi.nl, an authorized CRU data exploration app and repository. The black line shows the monthly mean, while the grey shaded area shows the 2.5%–97.5% range of the data. It is clear from the figure that a seasonal mean can remain below a given value (e.g., 30°C) while individual days can be much hotter.

Carleton et al. [29] argued that the temperature data they used were consistent with an increasing number of growing season days above 30°C and consequently an increasing number of years with maize harvest shortfalls. Importantly, as Carleton et al. [29] explained, increases in the mean summer temperature only have to approach the 30°C threshold in order for there to be an increased frequency of growing season days at or above the threshold. This is because daily temperatures are distributed around seasonal averages, which means increases in the seasonal average correspond to more individual days with even higher temperatures (see Fig 5 for an illustrative example). So, the growing season mean only needed to increase to around 29°C—about the increase observed in temperature reconstruction used in the present study—to produce several additional days above 30°C. Given the 1% per day decline observed in Lobell et al. [30], these additional overly-hot days can be expected to have led to significant maize yield declines.

Maize shortfalls, according to Carleton et al. [29], exacerbated regional conflict levels because they precipitated crises of legitimacy for the Classic Maya elite. Researchers have long thought that political legitimacy was crucial for the success of Classic Maya rulers [e.g., 62–68]. Rulers had to maintain legitimacy in the eyes of the general population, especially in the eyes of other elites and landholders with whom they formed political and economic alliances [63, 69–71]. Without their support, rulers would have been unable to command any power and consequently could no longer rule. Importantly for present purposes, successful maize harvests appear to have been an indicator of legitimacy [67, 72]. Rulers were responsible for ensuring successful crops and maize was particularly significant because it was tied to communal identity [72]. Many centres named in the epigraphic record contained references to local maize varieties and rulers’ names often contained references to the Classic Maya maize god [72]. Consequently, reduced maize yields or severe crop failures were likely perceived as ritual and political failures, which would have undermined political legitimacy. Carleton et al. [29] hypothesized that these crises of legitimacy exacerbated conflict among the Classic Maya: faced with a legitimacy problem, Classic Maya rulers would have sometimes sought to regain that lost legitimacy by waging war against other rulers. One consequence of this, Carleton et al. [29] argued, would have been a relationship between summer temperatures and conflict levels.

It should be noted that this hypothesis is not intended to be a full causal account of Classic Maya conflict. Obviously, it is not supposed to explain the autocorrelated conflict that forms the basis of the Retributive Violence model. But it also is not supposed to explain all the variation in the non-autocorrelated conflict in the epigraphic record. Conflicts likely happened for a variety of political and economic reasons that had nothing to do with maize yield reductions. For example, it is plausible that increases in population density may have led to more conflict [73]. In addition, there is reason to think that temperature-driven legitimacy crises may not have always resulted in conflict. Classic Maya rulers were connected in alliance networks based on conquest and marriages between elite lineages [20, 69], and these alliances were often headed by overlords [20]. Thus, an individual ruler faced with a legitimacy crisis may have had to convince their allies and possibly an overlord to permit them to attack another polity, or even join in the attack. Given this, the temperature-driven legitimacy crises proposed by Carleton et al. [29] should be thought of as a potentially important contributor to, rather than the sole cause of, the variation in Classic Maya conflict.

It is also important to keep in mind that there is a distinction between the putative mechanism and the statistical model. While the results of the analyses reported here are more consistent with Carleton et al.’s [29] hypothesis than Kennett et al.’s [28] one, this is only because the temperature reconstruction better predicts Classic Maya conflict levels than the rainfall proxy does. Our statistical results alone do not suggest any particular mechanism for the underlying relationship between the temperature reconstruction and the epigraphic proxy for Classic Maya conflict. Other mechanisms may be imagined that could fit the statistical evidence and such mechanisms should be explored. That said, any causal account of Classic Maya conflict needs to conform to the available evidence, and that evidence now includes our statistical results, which demonstrate that one of the most frequently cited proxies for past rainfall in the region—the Yok Balum Cave speleothem *δ*^18^O record—does not explain the available evidence for through-time variation in conflict levels, while the Cariaco Basin summer SST proxy does.

The failure of the analyses reported here to find evidence for an impact of rainfall variation on conflict levels is not unusual when we look at studies dealing with other regions. A number of such studies have found that rainfall variation—especially drought severe enough to impact agricultural productivity—either has no effect on conflict levels, or actually tamps down conflict (e.g, [10, 14, 11]). However, our finding regarding rainfall variation is still somewhat puzzling. The reason for this is that it is clear that the Classic Maya relied heavily on rainwater for both drinking and agriculture [66]. Given this, it seems reasonable to suppose that inter- and intra-annual variations in rainfall, including meteorological droughts and other anomalies, would have impacted crop yields, and that the variation in crop yields would have affected various aspects of economy and society in general, including inter-polity relationships [66, 67, 74–76]. Why, then, did we find an impact of temperature but not an impact of rainfall variation? Why weren’t both variables implicated in the changes in conflict levels?

So far, we have been able to identify three potential explanations. One is that the Yok Balum Cave record does not reflect economically relevant rainfall variation despite reflecting annual amounts. It has recently been shown that the Yok Cave *δ*^18*O*^ record is affected by storminess, especially hurricane activity [39], and it may also mask important seasonal variations in rainfall [77]. Thus, while the record we analysed may be highly correlated with past annual rainfall amounts, changes in cyclone activity or the seasonal distribution of rainfall are not clearly distinguishable from the overall amount effect. This means that the impact of two economically relevant rainfall-related phenomena on conflict may be obscured. Increases in storm activity would likely have affected crop yields because of the destructive power of cyclones and a redistribution of rainfall within a given year could have deprived crops of water during the growing season. Importantly, increasing SST in the Caribbean region has been shown to correlate with increasing cyclone activity which occurs seasonally over the summer months including the maize agricultural season [78, 79]. This means that increased storminess and temperature may have had a dual negative effect on crop yields. For these reasons, rainfall variation, storms, and drought (specifically agricultural drought) may still have affected conflict levels even though the annual rainfall proxy we analyzed does not predict variation in the conflict data. It should be possible to evaluate this hypothesis once the new record from Yok Balum Cave, which better isolates rainfall amount, is made publicly available [39].

Another potential explanation is that increases in temperature led to moisture shortages, which would mean that both water and heat were involved in the conflict generating process. As we explained earlier, recent research has found that even with adequate rainfall, increased temperatures negatively affect maize yields. At the same time, though, heat stress in maize is moderated by moisture availability and additional surface heat is known to lead to increased soil evapotranspiration [80]. Thus, it is possible that additional heat could have led to agricultural drought—insufficient soil moisture with a corresponding impact on agricultural productivity—absent any significant rainfall deficits. This process could have been exacerbated by Classic Maya landscape alterations, such as deforestation [81, 82], which could have made the soil even more susceptible to moisture loss. It is possible, therefore, that while rainfall shortages may not have affected conflict levels, water availability more generally may still have been involved. At the moment, though, we can only evaluate the potential impact of variations in yearly rainfall—i.e. meteorological drought—and not water shortages more generally. Devising ways of assessing the impact of the different types of drought on Classic Maya conflict should also be a priority for future research. Intensified terracing and a larger number of water management features might indicate an increase in concern about water shortages throughout the Classic period, and so such evidence could be used to separate out the effects of meteorological and agricultural droughts and build a more complex conflict model that accounts for the impact of water shortage more generally rather than just annual variation in rainfall.

The last potential explanation we have been able to identify is that the Classic Maya were well-adapted to rainfall shortfalls, but not to increases in temperature. Rainfall amounts and distribution throughout the year tend to vary a good deal on short time-scales in the Maya region [66]. These variations can sometimes be extreme, including meteorological drought [76, 83], and several studies have concluded that major decreases in precipitation occurred at various times in Classic Maya history, especially toward the end of the Classic Period [e.g., 77, 84, 85]. However, there is reason to think that the Classic Maya were generally able to cope with rainfall anomalies [66, 74, 75]. Agriculture has been practiced successfully in the region for thousands of years despite the marked variability in rainfall [62]. The Classic Maya made extensive use of terracing, employed natural and artificial reservoirs, and engaged in sophisticated hydroengineering projects, all of which are evidence of cultural adaptation to intra- and inter-annual variation in water availability [75, 83]. Of course, a particularly severe drought or change in seasonal distribution could have overwhelmed the Classic Maya attempts to buffer themselves against rainfall variation, leading to economic and social upheaval, perhaps even conflict in some cases [83, 86]. But long-term conflict levels may have been largely unaffected by rainfall variation because most of the variation was short-term and the Classic Maya were culturally adapted to it.

Temperature change, in contrast, may have presented a different challenge, one that the Classic Maya could not adapt to. The temperature record we analysed indicates very little short-term variation in regional temperatures. This pattern is similar to the pattern of temperature variation in the Maya region today. There is generally minimal intra- or inter-annual variation in temperature, as with most tropical zones [87]. Thus, the Classic Maya likely would not have been aware of the rising average temperature and therefore would not have developed cultural adaptations to temperature change. Furthermore, storing rainwater or diverting it around a field is undoubtedly much easier than artificially cooling crops. Thus, even if the need to adapt to temperature change were apparent to the Classic Maya, they may not have been able to do so despite their capacity for sophisticated water management. As with the previous two hypotheses, this suggestion will need to be tested in a future study.

Several other directions for future research suggest themselves. One is a more detailed analysis of past temperature variation. At the moment, there is too much uncertainty in the summer SST record to estimate relevant intermediate temperature variables, like the distribution of growing degree days above 30°C. We can say that the annual average temperature seems to have increased throughout the Classic period. But more detailed assessments of the distribution of temperature values and a more concrete linking-function connecting long-term temperature variation to conflict levels are required to properly understand the relationship between temperature, water availability, and conflict levels.

Another direction for future research entails developing proxies for maize yields. The hypothesis put forward by Carleton et al. [29] depends on a link between temperature and maize yields. Thus, proxies for past maize yields would be a very useful addition to the model and could be used to test the hypothesis more directly. At the moment, it is not entirely clear which potential proxies should be used, but perhaps they could be based on pollen frequencies, soil isotope records [88], or through-time changes in dietary isotopes indicative of maize consumption (e.g, [89, 90]). These more direct indicators will be necessary to determine whether the political legitimacy mechanism proposed by Carleton et al. [29] is consistent with the totality of evidence in the Classic Maya archaeological and historical records.

A third direction for future research concerns the evidence for conflict. While the trends in the epigraphic data we analysed appear to reflect trends in the archaeological evidence of violent conflict among the Classic Maya, it would be useful to generate a time-series that reflects through-time variation in Classic Maya conflict based on archaeological data—e.g., dates for the construction of defensive structures, frequency of weapons in dated artifact assemblages, and/or the prevalence of traumatic lesions on skeletal remains. When combined with the Bayesian method utilised here, such a time-series would enable a strong independent test of the rainfall and temperature hypotheses.

Lastly, it would be useful to delineate, and examine the impact of, extreme climatic events on conflict among Classic Maya polities. While our analysis identified no impact of trends in annual rainfall variation on Classic Maya conflict levels, individual extreme events—like severe droughts—might have caused short term fluctuations in conflict levels. This distinction between long-term variation and short-term extremes should be investigated, since it could lead to a better understanding of the climate-conflict process.

We will end by returning to where we began—the broader discussion about climate change and conflict. We now have results from analyses of the impact of temperature change on conflict over the long term for three areas—the Maya region (Carleton et al., 2017, this study), China (Zhang et al., 2006), and Europe (Carleton et al., 2021). Strikingly, the results in question encompass all the possible outcomes. While Carleton et al.’s (2017) analyses and the analyses presented here indicated that hotter temperatures led to more conflict among the Classic Maya between the 3rd century CE and the 10th century CE, Zhang et al.’s (2006) analyses suggested that cooler temperatures resulted in more conflict in China between the 11th century CE and the 20th century CE. Carleton et al.’s (2021) results were different again. They found no impact of temperature on conflict levels in Europe between the 11th century CE and the 20th century CE. These divergent results not only indicate that temperature does not have a simple, linear effect on conflict, contrary to what a number of authors have argued (e.g. Burke et al. 2009; O’loughlin et al. 2012; Maystadt et al. 2012); they also do not support Burke et al.’s (2015) suggestion that it is extreme temperatures—hot or cold—that result in increased conflict. Instead, the divergent results imply that the impact of temperature on conflict levels is context dependent: in some contexts increasing temperatures will likely drive up conflict; in others decreasing temperatures will result in more conflict; in still others changes in temperature will not affect conflict levels.

## Supporting information

S1 File This supplement contains information about the priors used in our model and MCMC diagnostics. It also describes our comparison between the Cariaco SST record and CRU data from the Maya region spanning the 20^*th*^ Century.
(ZIP)
