# Supplementary material for: Rainfall, temperature, and Classic Maya conflict: A comparison of hypotheses using Bayesian time-series analysis
Source: PLoS One. 2021 Jul 30;16(7):e0253043. doi: 10.1371/journal.pone.0253043 (PMC8323947; doi:10.1371/journal.pone.0253043)
Supplement: S1 File — It also describes our comparison between the Cariaco SST record and CRU data from the Maya region spanning the 20th Century. (ZIP) [file pone.0253043.s001.zip › MayaConflictRedux PLoS SI.pdf]

Supporting Information for Collard et al.'s 'Rainfall, temperature, and Classic Maya conflict: A comparison of hypotheses using Bayesian time-series analysis'

Mark Collard
Department of Archaeology
Simon Fraser University
Burnaby, BC, Canada
mcollard@sfu.ca

W. Christopher Carleton
Extreme Events Research Group
Max Planck Institutes for
Chemical Ecology,
The Science of Human History, and
Biogeochemistry
Jena, Germany
wcarleton@ice.mpg.de

David A. Campbell
School of Mathematics and Statistics
Carleton University
Ottawa, ON, Canada
davecampbell@math.carleton.ca

July 20, 2021

1 Raw Data Exploration

At the request of an anonymous reviewer, we have included a pairs plot of the raw data used in our analysis (see Fig. 1). It is important to note, however, that the relationships observable in this plot do not properly reflect the autocorrelation structure of the conflict record—this is fully accounted for in the time-series model we developed, as described in the main manuscript associated with this document. Thus, the plots alone can potentially be misleading and should be viewed with caution.

2 Setting the Priors

Establishing prior distributions is part of any Bayesian data analysis [1], and using informed priors is crucial for meaningful model comparison involving Bayes Factors [2]. In the present study, we aimed to use priors that would not lead to unrealistic predictions for the number of conflicts per monument. This criterion meant, for example, that extreme values for the regression coefficient should not lead to numbers of conflicts per monument much more extreme than

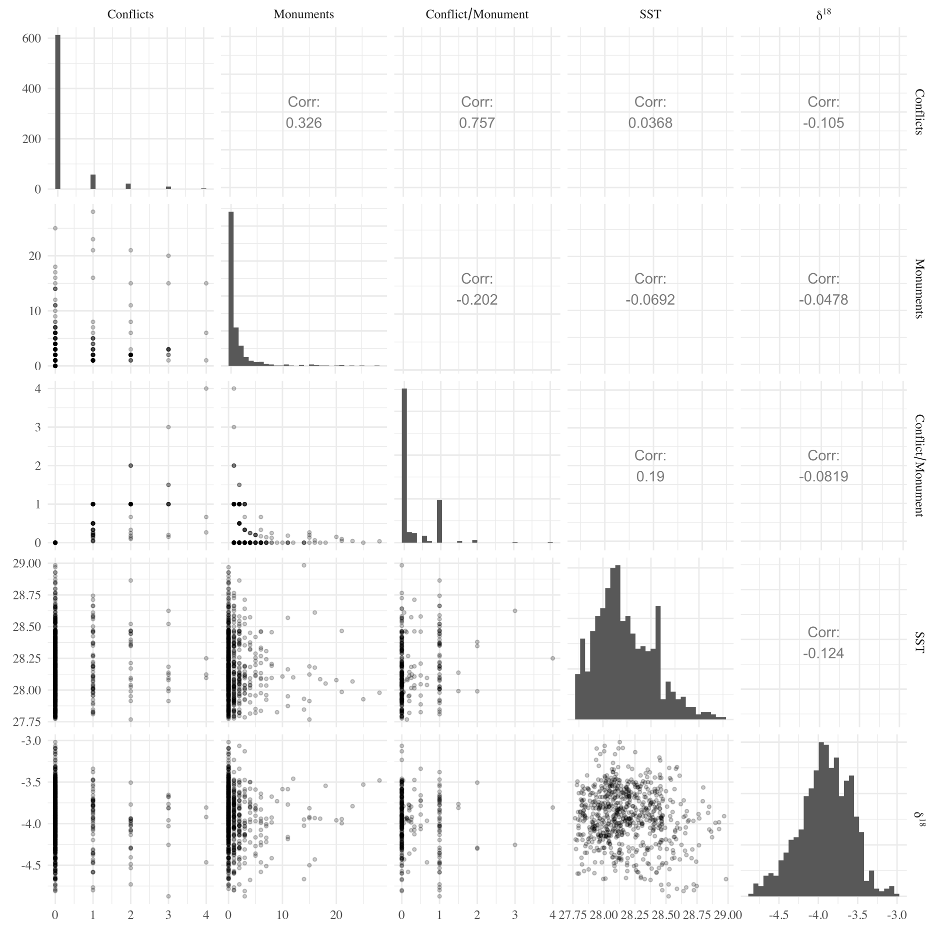

Figure 1: Pairs plot displaying the time-series data used in our analysis. The lower left triangle of the plot contains bivariate scatter plots; the diagonal contains raw, univariate histograms; and the upper right triangle displays Pearson's correlation values for each pair of time-series.

those observed in the data set. Practically, this meant choosing a variance for the regression coefficient priors that would not lead to absurd predictions of the number of conflicts per monument. To identify a sensible variance, we plotted three histograms. These included the number of conflicts per monument, the mean-centered Cariaco Basin summer temperature reconstruction time-series (Fig. 2a), and the mean-centered $\delta^{18}O$ rainfall/aridity proxy (Fig. 2b). We noted that for the vast majority of the conflict time-series, there were fewer than four conflicts per monument per year. So, we reasoned that five conflicts per monument was a sensible soft upper limit for the mean of the Poisson-distributed time-series variable. This line of reasoning led to the following inequality:

$$e^{\beta X_t} \lessapprox 5 \tag{2.1}$$

or equivalently,

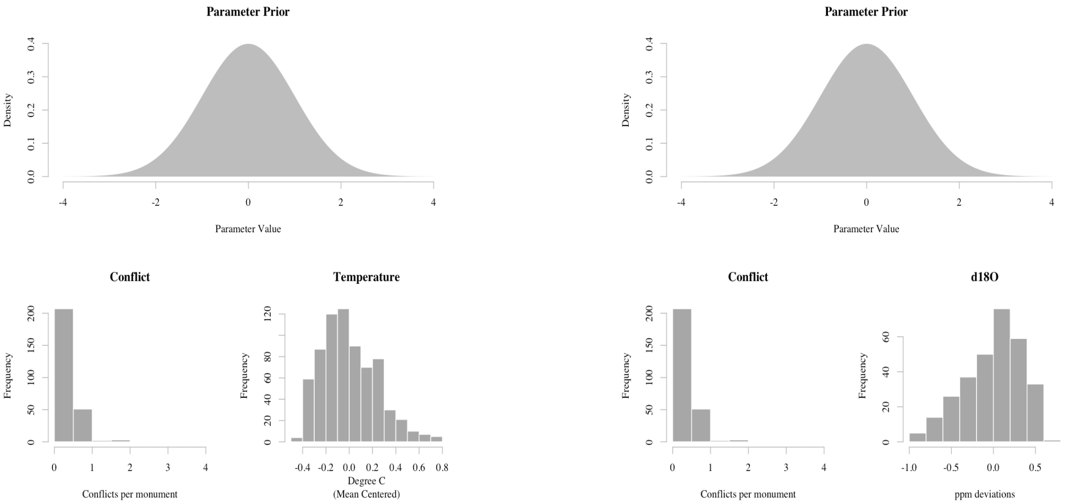

(a) Temperature Prior (b) Rainfall Prior

Figure 2: Distribution of mean-centered temperature, mean-centered $\delta^{18}O$, conflicts per monument, and the prior for the regression coefficients of each climate covariate.

$$\beta \lessapprox \frac{log(5)}{X_t} \tag{2.2}$$

To find a sensible top-end value for β we first needed to assess the scale (and range of possible values) for X_t. To do this, we examined the histograms for temperature and $\delta^{18}O$. We noted that the largest magnitude value for (mean-centered) temperature in the Cariaco Basin reconstruction was about 0.8 and the largest value for the $\delta^{18}O$ record was about 0.6. Given these values, we could plug reasonable top-end values for X_t into eq. 2.2:

$$\beta_{temperature} \lessapprox \frac{log(5)}{0.8} \tag{2.3}$$

$$\beta_{\delta^{18}O} \lessapprox \frac{log(5)}{0.6} \tag{2.4}$$

which led to approximate top-end values for β of 0.87 and 1.2, respectively. Using these values as a rough guide, we decided to use normally distributed priors with means of zero and standard deviations of 1.0. With standard deviations of 1.0, roughly 68% of the time β would have a magnitude (absolute value) less than 1.0. Such priors, we reasoned, would meet our criterion for sensible predictions about conflicts per monument while also being liberal enough to allow the data to provide accurate parameter estimates.

We used similar reasoning to establish priors for the other parameters in the model. With some of the other parameters, however, we used more agnostic priors—i.e. slightly larger variances—because the parameters in question were not the focus of our model comparisons and we had less domain information about their likely values.

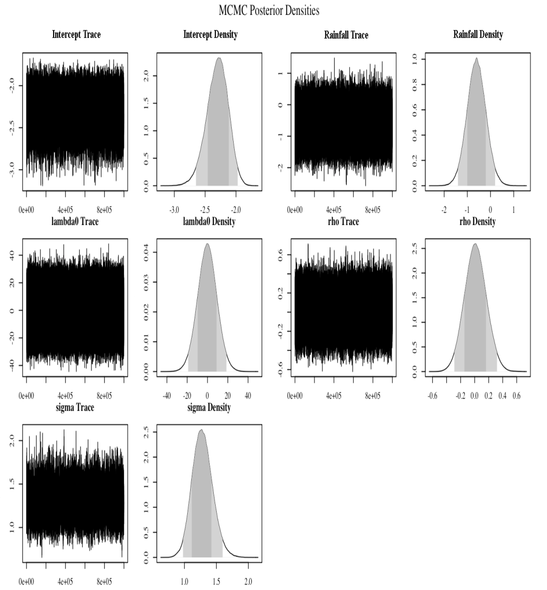

(a) Rainfall

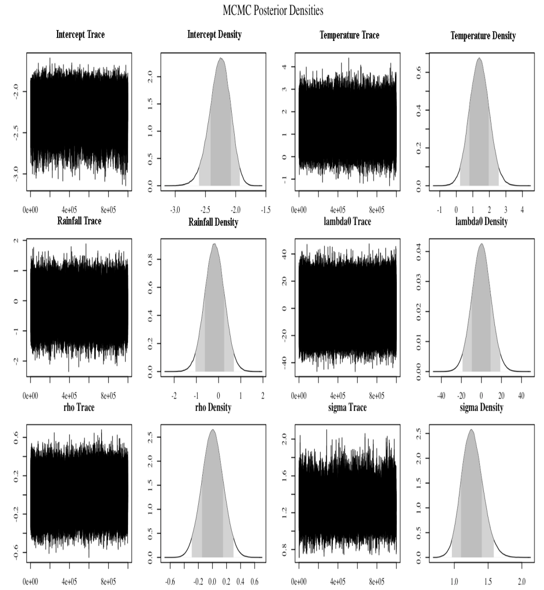

(b) Combined

Figure 3: Posterior densities and MCMC trace plots for the Rainfall and Combined models. The density functions contain two shaded regions: light grey corresponds to the 95% highest density region; and the dark grey corresponds to the 68% highest density region.

3 Model Posteriors and MCMC Trace Plots

As with the focal model, Temperature, we produced plots of the posterior distributions and MCMC chains (trace plots) for the other two models, Rainfall (Fig. 3a) and Combined (Fig. 3b). Since we used the Savage-Dickey method for estimating Bayes Factors with nested models, there was no need to estimate parameters for a separate Retributative model.

4 Convergence Diagnostics

With any MCMC-based Bayesian analysis it is important to ensure that the posterior distributions have been appropriately sampled. We used a combination of visual inspection (e.g., Fig. 3b and 3b) and a standard diagnostic called the Geweke statistic [3]. The Geweke statistic is essentially a t-test that compares the distribution of posterior MCMC samples from the first 10% of a given MCMC chain to the last 50% of the same chain. If the test finds no significant difference between the means of those distributions, then the MCMC algorithm has produced a long-run stationary sample of the relevant marginal posterior distribution. A stationary MCMC chain—often considered to have ”converged”—is likely an unbiased sample of the relevant posterior. The following table shows the Geweke statistics for the MCMC chains of the parameters for each of the three models we evaluated. The statistics indicate that the MCMC algorithm converged to unbiased samples of the posteriors in each case, further supporting the visual appearance of convergence in the relevant MCMC trace plots.

Table 1: Geweke statistics for MCMC chains of the parameters for each of the three models we analyzed. Each row contains statistics for a given model while the columns contain statistics for a given parameter MCMC chain. The statistics are reported in standard deviations of the standard normal distribution (rounded to the second decimal place), which means that only values larger in magnitude than approximately 2.5 would fall outside the 99% confidence interval. Since none of the values do, we concluded the MCMC chains were stationary for the parameters in each model, which in turn led us to believe the relevant posterior distributions were appropriately sampled.

Model	Intercept	Temp.	Rain.	λ_0	ρ	σ
Temperature	0.49	0.51		0.36	-0.92	-0.50
Rainfall	-0.13		-0.09	-1.06	0.61	0.34
Combined	0.93	0.02	0.63	1.42	-1.8	-0.16

5 Exploring More Agnostic Priors

It is generally considered best practice to use informed priors whenever possible in a Bayesian analysis [4, 2]. This is particularly true of studies in which competing models are compared with Bayes Factors because Bayes Factors are known to be sensitive to priors [2]. As a corollary, however, it is also important to explore the impact of using alternate priors to determine how sensitive a given finding is to changes in prior information and/or assumptions. With this in mind, we re-ran our analyses with ”looser” priors for β since our findings and subsequent interpretations depend on the Bayes Factors we estimated from that parameter. Our initial prior for β was normally distributed with a mean of zero and standard deviation of 1.0. For this sensitivity analysis we increased the standard deviation to 10.0. To be clear, such a wide prior implies that increases in temperature like the ones we observe in the Cariaco reconstruction could conceivably yield a mean level of $e^{\beta X_t} = e^{20 X_t} = e^{(20)(0.8)} = e^{(20)(0.8)} \approx 8,886,110.5$ conflicts per monument, which is clearly an unrealistic number. Such a wide prior would also lead to very conservative Bayes Factors because only very large effects would shift our beliefs about the putative climate-conflict relationship away from the null. Still, relaxing the prior in this way allowed us to explore whether our weakly informative priors substantially biased our results.

The results of the sensitivity analysis agree with our primary findings. After running a new set of analyses with much wider priors, we found that the Temperature model was more probable given the data than the Retributative model and the other two models. While the Bayes Factors were predictably more conservative, the overall result was the same. The means of the posterior parameter estimates were also nearly identical those we obtained previously. Thus, the weakly informative priors we used for our primary analysis did not introduce any significant biases in terms of either our model comparisons or our parameter estimates.

Table 2: Model results given vague priors. Rows represent the primary results of a single model. The first column indicates which if any climate covariates were included in a given model: "Retributative" indicates a retributive conflict-only model; "Temperature" a model containing the temperature covariate; and so on. The third column contains the Bayes Factors, which indicate the weight of evidence for each model in a given time period relative to the baseline, Retributive model. The Bayes Factors can be straightforwardly interpreted as the number of times more probable a given model is than the Retributive model. The last two columns contain the mean of the posterior density of the regression coefficient estimated for a given climate covariate.

Model	Bayes Factor	Temp. Coef.	Rain. Coef.
Retributive	1		
Temperature	2.08	1.45	
Rainfall	0.12		-0.6
Combined	0.09	1.39	-0.18

6 Correlation between Cariaco summer SST and Maya region near-surface Temperatures

6.1 Objective

Compare the Cariaco SST record we analyzed to Climate Research Unit (CRU) temperature data for the instrumental period covered by both data sets.

6.2 Get CRU Data

The CRU provides access to a global gridded temperature record. These records can be accessed (after registration) at `https://catalogue.ceda.ac.uk/`. For more information about the data, see Harris, I., Osborn, T.J., Jones, P. et al. Version 4 of the CRU TS monthly high-resolution gridded multivariate climate dataset. Sci Data 7, 109 (2020). https://doi.org/10.1038/s41597-020-0453-3.

The Center for Environmental Data Analysis (CEDA) hosts an online data repository and search service that we used to extract the CRU temperature data for the Maya region. The subsetting tool is particular useful since it can be used to select a region and time period subset from the total CRU gridded dataset (see figure below).

We selected an area roughly covering the Classic Maya region and downloaded the corresponding monthly gridded temperature data. These data are provided in multiple formats. We selected .csv format (See the ../Data folder of this archive).

Once obtained, we loaded the .csv data into an R dataframe:

```
d <- dir("../Data/CRU/")
d <- d[grep("tmp",d)]
cru <- data.frame()
for(j in d){
    f <- paste("../Data/CRU/",j,sep="")
    cru_tmp <- read.csv(f,head=F,skip=44)
```

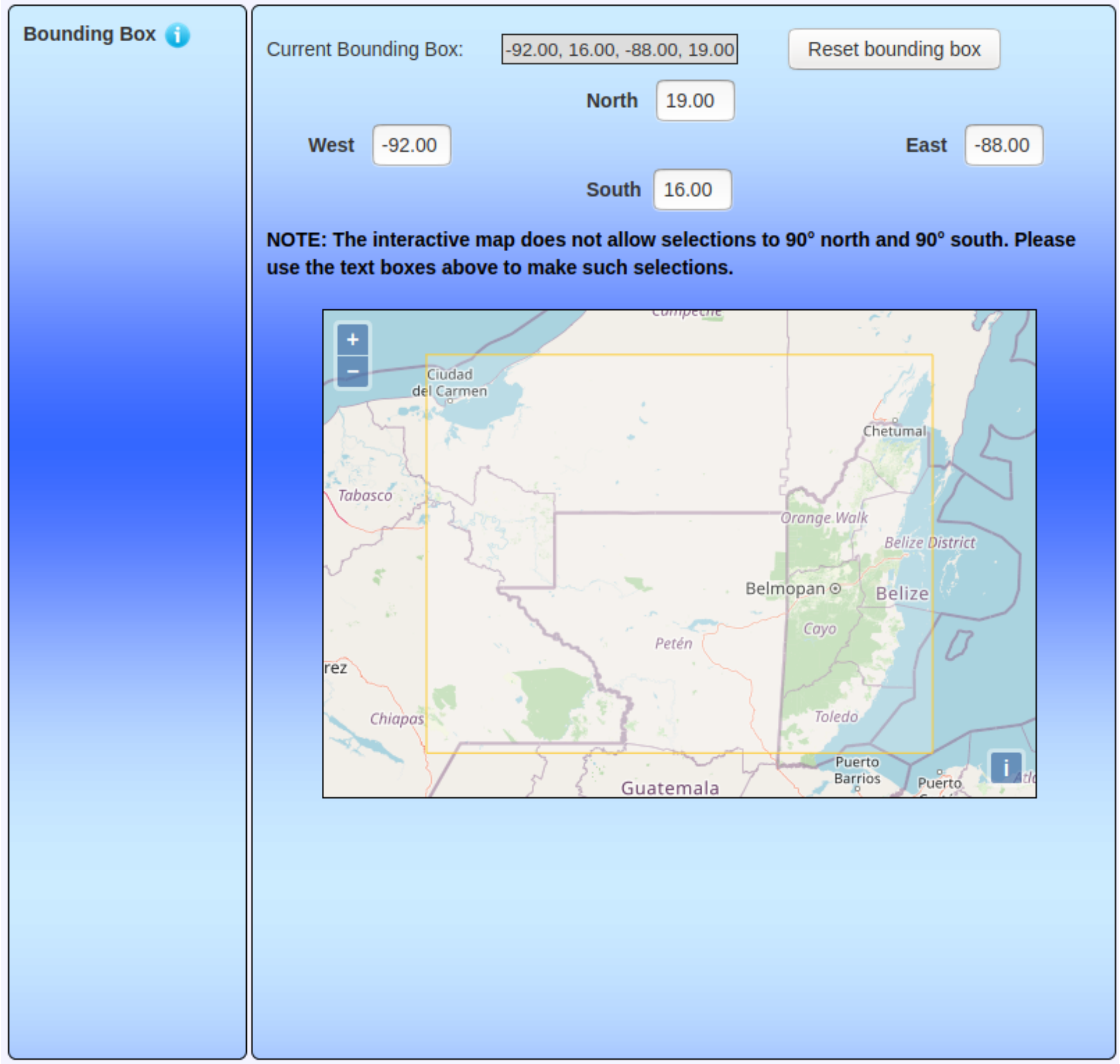

Figure 4: CEDA Subsetter. Note the bounding box we selected covers most of the Central Maya Lowlands, the core region where most of the conflict records were discovered.

```
    cru <- rbind(cru,cru_tmp)
}
head(cru)
```

```
##                V1    V2   V3   V4   V5   V6   V7   V8   V9
## 1 Data section 380.0   NA   NA   NA   NA   NA   NA   NA
## 2 Data section  17.4 21.7 23.1 23.5 22.3 21.1 22.6 24.2
## 3 Data section  20.1 21.0 22.7 23.2 22.1 20.7 19.9 22.9
## 4 Data section  21.6 22.3 22.9 22.9 22.0 21.9 22.0 22.8
## 5 Data section  23.3 22.9 22.6 22.4 21.8 22.0 22.6 23.1
## 6 Data section  23.2 23.0 22.5 22.2 21.6 21.4 22.3 23.0
```

A bit of cleaning is required to remove non-data:

```
cru <- as.matrix(cru[,-1])
cru <- cru[apply(cru,1,function(x)all(!is.na(x))),]
head(cru)
```

```
##        V2   V3   V4   V5   V6   V7   V8   V9
## [1,] 17.4 21.7 23.1 23.5 22.3 21.1 22.6 24.2
## [2,] 20.1 21.0 22.7 23.2 22.1 20.7 19.9 22.9
## [3,] 21.6 22.3 22.9 22.9 22.0 21.9 22.0 22.8
## [4,] 23.3 22.9 22.6 22.4 21.8 22.0 22.6 23.1
## [5,] 23.2 23.0 22.5 22.2 21.6 21.4 22.3 23.0
## [6,] 23.3 23.1 22.5 22.1 21.5 21.5 22.1 22.7
```

Next, we spatially averaged the CRU temperature data for the region and created two temperature time series spanning the overlap between the CRU data and the Cariaco SST reconstruction (i.e, 1901–2008). For one time series, we averaged the monthly data to produce an annual average temperature record. For the other, we included only the months corresponding to the summer SST record, namely June, July, and August, which produced an annual summer average record.

To create the time-series, we first need to generate spatial averages. The rows in the cru matrix each correspond to a latitude in the gridded dataset while the columns correspond to longitudes. There are 6 x 8 of these grid squares in our selected study area. Since the raw data are monthly temperatures, there are 12 of these 6-row chunks per year. Each chunk needs to be averaged to produce the spatial average. So, we will walk over the cru matrix in groups of 6 rows and calculate the average of all grid-cell values in that chunk (i.e., an average of all the values in each 6 x 8 grid):

```
month_chunks <- seq(1,dim(cru)[1],6)
monthly_means <- c()
for(j in month_chunks){
    m_tmp <- mean(cru[j:(j+5),])
    monthly_means <- c(monthly_means,m_tmp)
}
```

With a vector of monthly spatial means, we can now produce both time-series. First we produce the annual series by simply averaging every 12 elements in the `monthly_means` vector and then only the rows corresponding to June, July, and August. The data we downloaded actually corresponds to the latest CRU data and so spans 1901–2020 CE. So, we can also remove the elements corresponding to recent years not covered by the Cariaco SST reconstruction.

```
monthly_mean_matrix <- matrix(monthly_means,nrow=12)
annual_means <- colMeans(monthly_mean_matrix)[1:108]
summer_means <- colMeans(monthly_mean_matrix[c(6,7,8),])[1:108]
```

6.3 Compare the records

We then compared these two time-series to the Cariaco summer SST record (SSTRub) with a simple linear regression.

First, we need to load the Cariaco SST record:

```
sst <- read.csv("../Data/wurtzel2013_CariacoSST.csv")
sst_1901_2008 <- subset(sst,YearCE >= 1901 & YearCE <= 2008)
```

Then, we can aggregate all three time-series into a dataframe:

```
tempdata <- data.frame(
                Year=1901:2008,
                CariacoSST=sst_1901_2008$SSTRub,
                CRU_Annual=annual_means,
                CRU_Summer=summer_means)
```

With this dataframe, we can now run a couple of simple linear regressions and store the results for further analysis and plotting:

```
glm_annual <- glm(CRU_Annual~CariacoSST,data=tempdata)
glm_summer <- glm(CRU_Summer~CariacoSST,data=tempdata)
```

To plot the model results, we used ggplot2.

```
library(ggplot2)
library(ggpubr)

pred_annual <- predict(glm_annual, se.fit=T)
pred_summer <- predict(glm_summer, se.fit=T)

pred_annual_df <- data.frame(
                    CariacoSST=tempdata$CariacoSST,
                    GLMFit=pred_annual$fit,
                    GLMSE=pred_annual$se.fit)
```

```
pred_summer_df <- data.frame(
                    CariacoSST=tempdata$CariacoSST,
                    GLMFit=pred_summer$fit,
                    GLMSE=pred_summer$se.fit)
```

6.3.1 Time Series

As always, it's useful to look at the raw data, which in this case are the three temperature time series:

```
ggplot(tempdata) +
    geom_path(mapping=aes(y=CRU_Annual,x=Year),colour="#ffbe0f") +
    geom_text(
            x=2000,
            y=mean(tempdata$CRU_Annual),
            colour="#ffbe0f",
            label="CRU Annual") +
    geom_path(mapping=aes(y=CRU_Summer,x=Year),colour="#bd2000") +
    geom_text(
            x=2000,
            y=mean(tempdata$CRU_Summer),
            colour="#bd2000",
            label="CRU Summer") +
    geom_path(mapping=aes(y=CariacoSST,x=Year),colour="#8c0000") +
    geom_text(
            x=2000,
            y=mean(tempdata$CariacoSST),
            colour="#8c0000",
            label="Cariaco SST") +
    labs(y="Temperature (C)", x="Time (CE)") +
    theme_minimal() +
    theme(text = element_text(family="Times", size=12))
```

6.3.2 Bivariate Plots and GLM Results

Then, we can look at each of the two CRU temperature series compared to the Cariaco reconstruction with the glm results plotted over top:

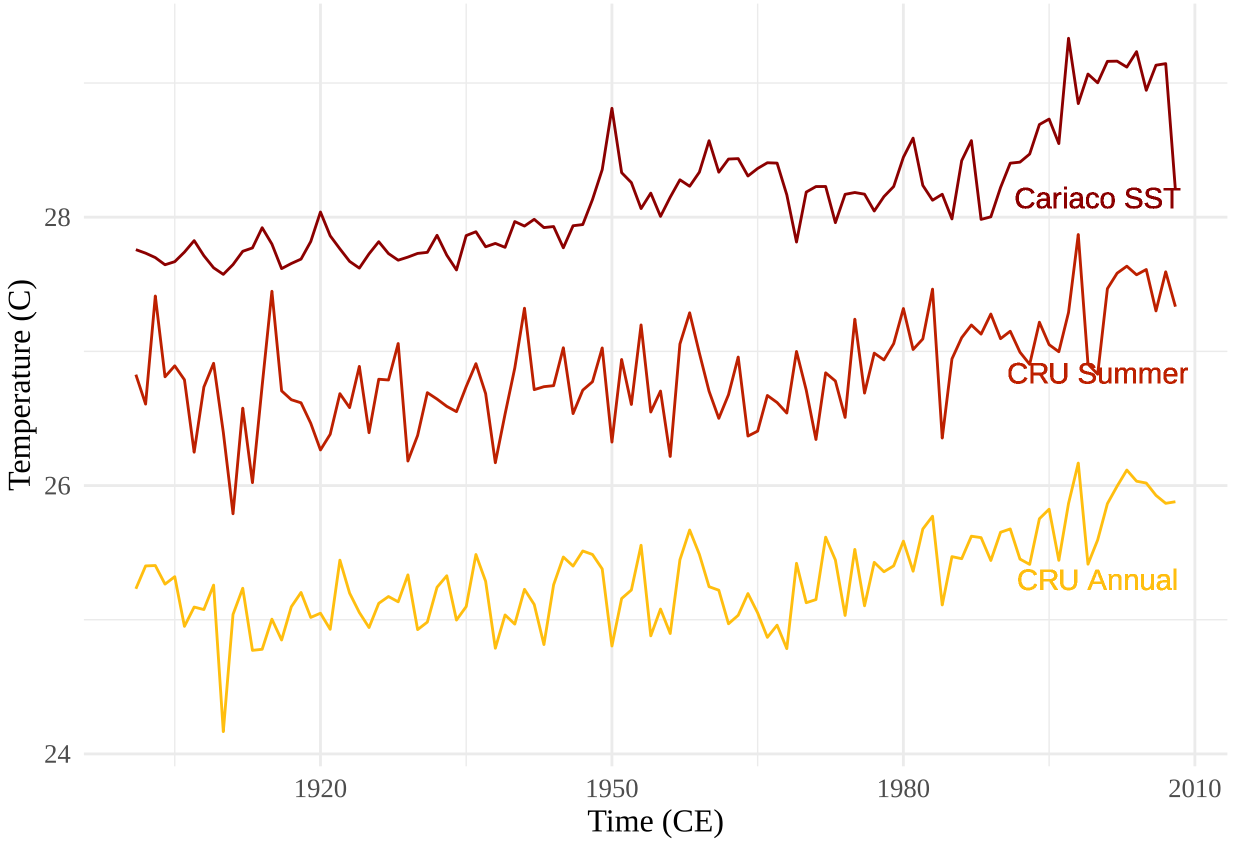

Figure 5: Temperature Time Series

```
p1 <- ggplot(tempdata) +
    geom_point(mapping=aes(y=CRU_Annual,x=CariacoSST),
        alpha=0.5) +
    geom_path(
        data=pred_annual_df,
        mapping=aes(y=GLMFit,x=CariacoSST),
        alpha=0.8
        ) +
    geom_ribbon(
        data=pred_annual_df,
        mapping=aes(x=CariacoSST,ymin=GLMFit - GLMSE*2.96,ymax=GLMFit + GLMSE*2.96),
        fill="steelblue",
        alpha=0.5
        ) +
    labs(y="Maya Region T (Annual)", x="Cariaco T") +
```

```
    theme_minimal() +
    theme(text = element_text(family="Times", size=12),
        axis.title.x=element_blank(),
        axis.text.x=element_blank(),
        axis.ticks.x=element_blank())

p2 <- ggplot(tempdata) +
    geom_point(mapping=aes(y=CRU_Summer,x=CariacoSST),
        alpha=0.5) +
    geom_path(
        data=pred_summer_df,
        mapping=aes(y=GLMFit,x=CariacoSST),
        alpha=0.8
        ) +
    geom_ribbon(
        data=pred_summer_df,
        mapping=aes(x=CariacoSST,ymin=GLMFit - GLMSE*2.96,ymax=GLMFit + GLMSE*2.96),
        fill="steelblue",
        alpha=0.5
        ) +
    labs(y="Maya Region T (Summer)", x="Cariaco T") +
    theme_minimal() +
    theme(text = element_text(family="Times", size=12))

ggarrange(p1,p2,
    ncol=1,
    nrow=2,
    align="v")
```

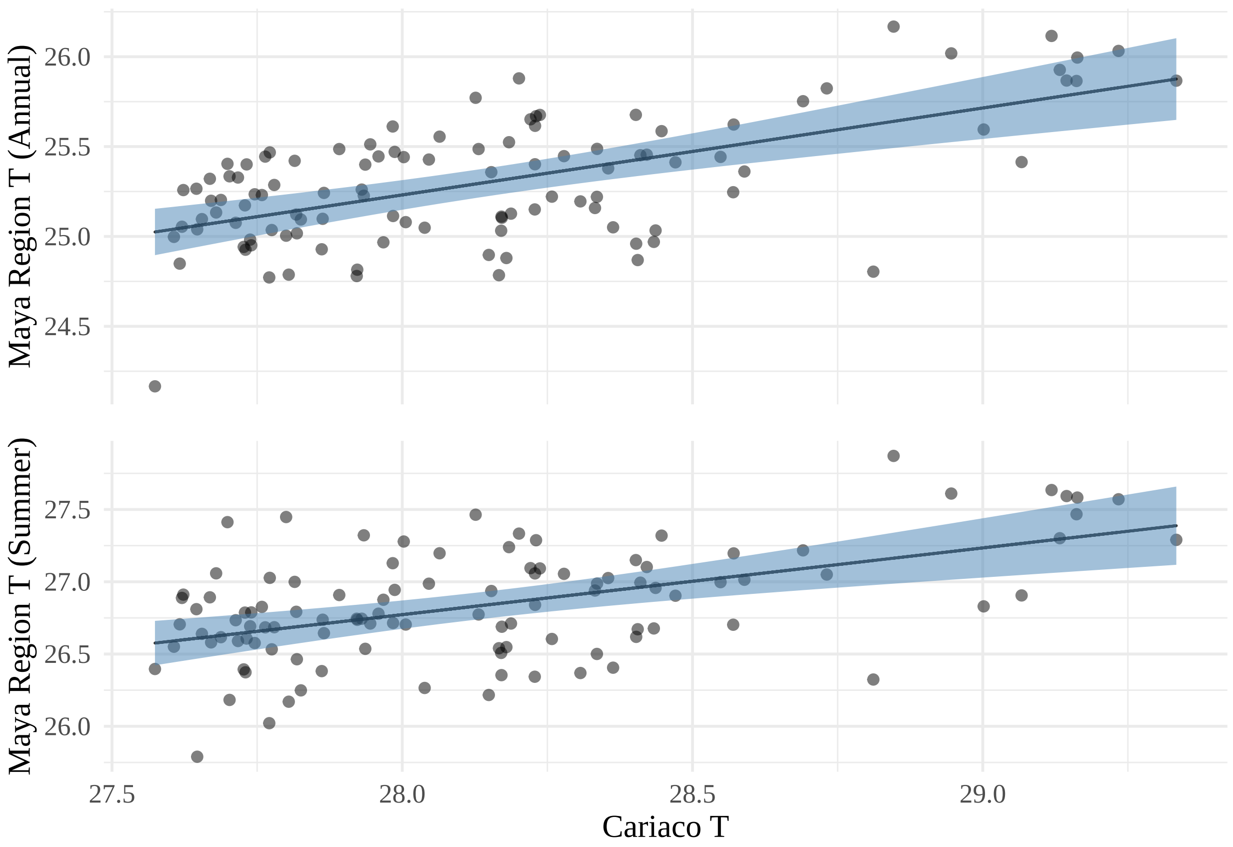

6.4 Effects

We can also display the regression results numerically.

6.4.1 Annual GLM

```
summary(glm_annual)
```

```
##
## Call:
## glm(formula = CRU_Annual ~ CariacoSST, data = tempdata)
##
## Deviance Residuals:
##      Min        1Q    Median        3Q       Max
## -0.85884  -0.17676   0.03006   0.20687   0.55137
##
## Coefficients:
##             Estimate Std. Error t value Pr(>|t|)
```

```
## (Intercept)  11.6871     1.7087   6.840 5.27e-10 ***
## CariacoSST    0.4837     0.0607   7.968 1.95e-12 ***
## ---
## Signif. codes:  0 '***' 0.001 '**' 0.01 '*' 0.05 '.' 0.1 ' ' 1
##
## (Dispersion parameter for gaussian family taken to be 0.07527857)
##
##     Null deviance: 12.7594  on 107  degrees of freedom
## Residual deviance:  7.9795  on 106  degrees of freedom
## AIC: 31.124
##
## Number of Fisher Scoring iterations: 2
```

6.4.2 Summer GLM

```
summary(glm_summer)
```

```
##
## Call:
## glm(formula = CRU_Summer ~ CariacoSST, data = tempdata)
##
## Deviance Residuals:
##      Min        1Q    Median        3Q       Max
## -0.82356  -0.21154   0.01089   0.19201   0.77889
##
## Coefficients:
##             Estimate Std. Error t value Pr(>|t|)
## (Intercept) 13.84829    2.03422   6.808 6.16e-10 ***
## CariacoSST   0.46158    0.07227   6.387 4.59e-09 ***
## ---
## Signif. codes:  0 '***' 0.001 '**' 0.01 '*' 0.05 '.' 0.1 ' ' 1
##
## (Dispersion parameter for gaussian family taken to be 0.1066938)
##
##     Null deviance: 15.662  on 107  degrees of freedom
## Residual deviance: 11.310  on 106  degrees of freedom
## AIC: 68.79
```

```
##
## Number of Fisher Scoring iterations: 2
```

References

1. Gelman A, Carlin JB, Stern HS, Dunson DB, Vehtari A, Rubin DB. Bayesian Data Analysis. 3rd ed. Boca Raton: CRC Press; 2014.
2. Wagenmakers EJ, Lodewyckx T, Kuriyal H, Grasman R. Bayesian hypothesis testing for psychologists: A tutorial on the Savage-Dickey method. Cognitive Psychology. 2010;60(3):158–189. doi:10.1016/j.cogpsych.2009.12.001.
3. Geweke J. Evaluating the Accuracy of Sampling-Based Approachesto the Calculation of Posterior Moments. In: Bernardo JM, Berger JO, Dawid AP, Smith AFM, editors. Bayesian Stastistics. 4th ed. Oxford: Clarendon Press; 1992. p. 169–193.
4. Gabry J, Simpson D, Vehtari A, Betancourt M, Gelman A. Visualization in Bayesian workflow. Journal of the Royal Statistical Society Series A: Statistics in Society. 2019;182(2):389–402. doi:10.1111/rssa.12378.
